# Utilising Inertial Measurement Units and Force–Velocity Profiling to Explore the Relationship Between Hamstring Strain Injury and Running Biomechanics

**DOI:** 10.3390/s25051518

**Published:** 2025-02-28

**Authors:** Lisa Wolski, Mark Halaki, Claire E. Hiller, Evangelos Pappas, Alycia Fong Yan

**Affiliations:** 1 Sydney School of Health Sciences, Faculty of Medicine and Health, The University of Sydney, Sydney, NSW 2050, Australiamark.halaki@sydney.edu.au (M.H.); claire.hiller@sydney.edu.au (C.E.H.); 2 School of Health and Biomedical Sciences, Royal Melbourne Institute of Technology, Melbourne, VIC 3000, Australia; evangelos.pappas@rmit.edu.au

**Keywords:** sensor, measurement, accelerometer, gait, biomechanics, sprint, kinematic, kinetic, spatiotemporal

## Abstract

The purpose of this study was to retrospectively and prospectively explore associations between running biomechanics and hamstring strain injury (HSI) using field-based technology. Twenty-three amateur sprinters performed 40 m maximum-effort sprints and then underwent a one-year injury surveillance period. For the first 30 m of acceleration, sprint mechanics were quantified through force–velocity profiling. In the upright phase of the sprint, an inertial measurement unit (IMU) system measured sagittal plane pelvic and hip kinematics at the point of contact (POC), as well as step and stride time. Cross-sectional analysis revealed no differences between participants with a history of HSI and controls except for anterior pelvic tilt (increased pelvic tilt on the injured side compared to controls). Prospectively, two participants sustained HSIs in the surveillance period; thus, the small sample size limited formal statistical analysis. A review of cohort percentiles, however, revealed both participants scored in the higher percentiles for variables associated with a velocity-oriented profile. Overall, this study may be considered a feasibility trial of novel technology, and the preliminary findings present a case for further investigation. Several practical insights are offered to direct future research to ultimately inform HSI prevention strategies.

## 1. Introduction

Hamstring strain injury (HSI) is of considerable concern in high-speed running sports. With a particularly high incidence [1,2,3,4,5,6] and recurrence rate [1,2], HSI presents a significant challenge for clinicians and coaching staff. Age and previous history of HSI are well-established risk factors for HSI [3], but several other factors are considered to play a role, including high-speed running exposure [4], reduced flexibility [5], and decreased hamstring strength metrics [3]. While the role of the hamstrings in high-speed running is well researched and understood [6], the connection between running biomechanics and HSI remains less clear. This connection has been studied in two reviews [7,8], with both concluding emerging evidence suggests an association between running biomechanics and HSI, but more research is needed.

Kinematic experimental studies suggest lumbopelvic control, anterior pelvic tilt, forward trunk lean, trunk lateral flexion, and maximal hip flexion angle may be linked to HSI [9]. From a kinetic perspective, decreased horizontal propulsive force is suggested to be associated with HSI [7]. The hamstrings play a major role in horizontal force production during high-speed running [10], making a compelling case for reduced horizontal force as a cause and/or consequence of HSI [11,12,13,14]. Further field-based evaluation of these variables is required to define thresholds for injury screening out of the lab environment, and subsequently inform prevention strategies.

The gold standard for biomechanical analysis is the Motion Analysis System (MAS). Although the MAS provides exceptional detail on spatiotemporal, kinematic, and kinetic biomechanical metrics, environmental constraints hinder its ability to accurately replicate movement in real-world contexts [6]. The 21st century has seen the introduction and development of various innovative field-based technologies, such as vision-based measurement systems [15,16,17,18], inertial measurement units (IMUs) [19,20,21], optical fibre technology [22,23], pressure sensing insoles [24,25], and force–velocity profiling (FVP) [26,27]. To investigate the aforementioned kinematic and kinetic variables of interest in a field-based setting, portable technology that is validated for use in high-speed running is required. Additionally, the technology should be relatively low-cost to enable adoption by sporting organisations for potential injury prevention initiatives. For the purposes of this study, IMU and FVP systems were considered the most appropriate field-based technologies.

Inertial measurement units (IMUs) are a growing, low-cost, portable technology for quantifying running biomechanics [28]. IMUs include three components: an accelerometer, a gyroscope, and a magnetometer, which provide measurements of three-dimensional linear acceleration, angular velocity, and orientation. Multiple calibrated IMUs can track joint angles, enabling kinematics analysis during functional activities [29,30]. Although IMUs can produce kinetic outputs through computational methods, their accuracy in predicting ground reaction forces is variable [31,32]. A recently published study validated an IMU system against the MAS for use in high-speed running [33]. The IMU system correctly identified the point of contact (POC), which was subsequently used to calculate stride time, and accurately measured the sagittal plane pelvic tilt and hip angle at the POC [33]. Unfortunately, the validation study found the IMU system to be inaccurate and inconsistent in measuring knee flexion and shank angles at the POC, making it unsuitable for such measurements [33]. The current study aimed to use the same IMU system in the field in order to retrospectively and prospectively analyse the relationship between validated outcomes; namely sagittal plane pelvis and hip kinematics at the POC, and HSI.

Sprint force–velocity profiling (FVP) is an innovative approach to characterising the kinetics of acceleration and sprint mechanical performance. The relationship between running velocity and the ability to generate horizontal force on the ground is described by the linear force–velocity model. Given that power results from force multiplied by velocity, the slope of the force–velocity relationship displays the contributions of force and velocity in achieving maximum horizontal power output [26,27]. Although the time over a given sprint distance may be the same between two athletes, their FVPs may be different, e.g., higher force or velocity orientation [34]. In the last decade, a simple computational method for determining FVP using only anthropometric and spatiotemporal data was validated against a track embedded with force plates [35]. Initially, high-speed digital cameras, radar technology, and timing gates were used to calculate times and velocity [35,36,37,38]. Shortly after, a simple iPhone application calculating split times for FVP modelling was also validated [39]. A secondary aim of our study was to utilise the iPhone application method in the field to retrospectively and prospectively analyse the relationship between FVP (as a representation of horizontal force production) and HSI.

The overall purpose of this study was to explore associations between running biomechanics and hamstring strain injury (HSI) using field-based technology. We hypothesised that running biomechanics, namely spatiotemporal and kinematic variables as measured by an IMU system and FVP through an iPhone application, may significantly differ between participants with a history of HSI and controls. Retrospective and prospective analysis via a one-year injury surveillance will further inform which biomechanics variables may be a cause or consequence of HSI. Additionally, we aimed to offer ‘lessons learnt’ in using these novel technologies to direct future research.

## 2. Materials and Methods

### 2.1. Testing Protocol

The academic institution’s Human Research Ethics Board approved this protocol (project number 2018/674). Athletes aged 17–50 years who actively participated in a sport involving sprinting and did not have a current medical condition or injury that affected their ability to sprint pain-free were invited to participate in the study. Twenty-three amateur sprinters were recruited to perform a single maximum-effort 40 m sprint (mean age 25.7 ± 10.6 years; height 1.76 ± 0.08 m; weight 72.1 ± 10.1 kg; 10 females, 13 males). A significantly larger sample size was originally planned (planned: G-power output based on power = 0.8, n = 109), including various professional rugby, football, and soccer clubs. Unfortunately, the occurrence of the COVID-19 pandemic drastically limited the ability to test and recruit. Therefore, the study reported here is a feasibility study aimed at providing practical insights and directing future research to ultimately inform HSI prevention strategies.

Testing was conducted individually over a two-week period (based on participant availability). Following trial familiarisation (both verbally and through written participant information), participants provided informed consent and baseline details (age, sex, height, weight, HSI history).

**Figure 1:**
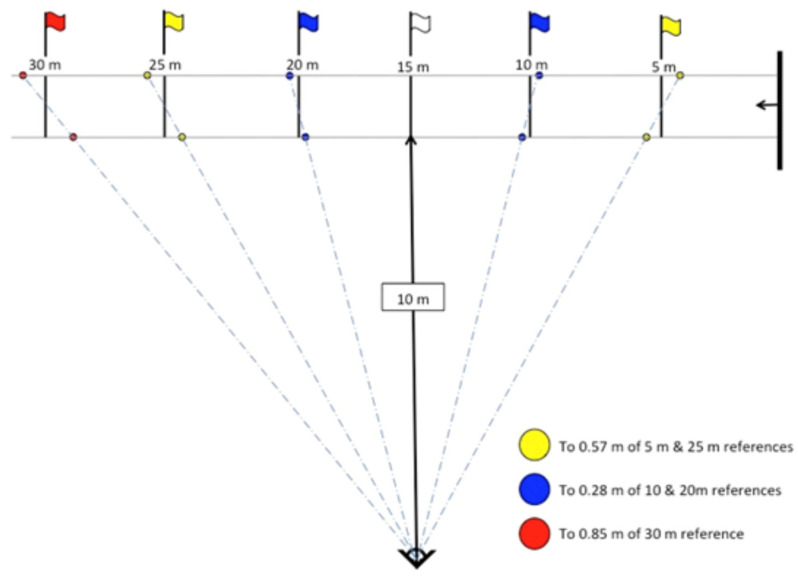
Force–velocity profiling setup taken from ‘MySprint’ iPhone application instructions (Version 1.10 installed on iPhone XR running iOS 13). Distances of 5, 10, 15, 20 and 30 metres are marked by flags.

A 40 m track was set up on grass with a line of tape measure and large blue markers denoting start and finish. The FVP protocol was conducted in accordance with ‘MySprint’ iPhone application instructions (Version 1.10 installed on iPhone XR running iOS 13). Tall yellow agility poles were used to denote 5 m intervals for the first 30 m. Poles were offset to account for parallax error (as per Figure 1). Sprint trials were filmed with the iPhone’s built-in camera on ‘Slo-mo’ for 1080 p at 240 fps, mounted on a tripod 10 m from the track enabling smooth rotational movement.

Five Noraxon ‘MyoMOTION’ IMUs and software (Noraxon USA Inc., Scottsdale, AZ, United States, Model 680 receiver, Model 610 sensor, MR 3.16 software) were used in this study for kinematic data capture during the steady-state phase of the 40 m sprint. The IMUs had >8 h operating time (3 h to recharge), were 37.6 mm × 52 mm × 18.1 mm in size, weighed 34 g, had a sampling rate of 200 Hz, a gyroscope speed of 2000 deg/s and an acceleration range of 16G, with ±1 degree in the sagittal and frontal plane and ±2 degrees in the transverse plane. The IMU system was previously validated against the gold standard MAS. The current setup replicated that of the validation [33].

IMUs were secured bilaterally to participants’ shanks and thighs with double-sided tape (Logemas Pty Ltd., Albion, Australia) and an elastic adhesive bandage (Elastoplast, Beiersdorf Australia Pty Ltd., North Ryde, Australia). The shank sensors were positioned laterally approximately 3–4 cm above the lateral malleolus and thigh sensors on the mid-lateral thigh. The final IMU was secured centrally on the sacrum via a Noraxon myoMOTION pelvic strap and reinforced with rigid tape (Elastoplast, Beiersdorf Australia Pty Ltd., North Ryde, Australia). The serial numbers of each IMU were input into the Noraxon software against the relevant body part where the sensor was placed. Then, body-to-sensor calibration of the IMUs was performed in the participants’ neutral standing posture.

**Figure 2:**
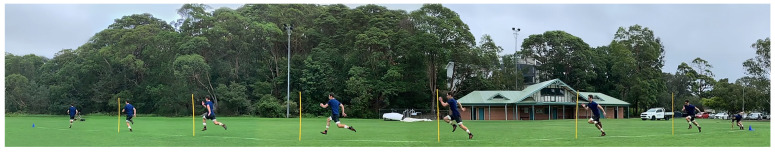
Photographic example of single maximum-effort 40 m sprint.

Following the FVP and IMU setup, each participant was instructed to complete a self-directed warm-up which generally consisted of graduated running, dynamic stretches, drills, and trial practice. The 40 m sprint trial (encompassing both IMU and FVP capture) was subsequently conducted on grass from a crouch start (see Figure 2). Air pressure (hPa) and temperature (°C) were recorded at the time of testing with iPhone XR running iOS 13 (Apple Weather application).

### 2.2. Surveillance Procedure

After the sprint trial, participants were followed for a one-year injury surveillance period, involving weekly mobile phone text message reporting through a survey administration platform (Qualtrics 2019–2020, Provo, UT, USA). Participants were asked the following: ‘In the last week, have you had pain in the hamstring region (back of thigh) that has affected your ability to train or compete?’ The text message required a ‘yes’ or ‘no’ response. If participants did not respond to the weekly text, they were sent a reminder text message. If after two reminder text messages, participants still did not respond, they were contacted by one of the study investigators to confirm ongoing study participation.

If the participant answered ‘yes’, a physiotherapist was notified to contact the participant to arrange an appropriate time/day/location to assess the injury free of charge. This physiotherapist conducted an initial assessment only and did not provide treatment. Further physiotherapy treatment was at the discretion of the participant. If the participant sustained an HSI and wanted to report it sooner than when the weekly text message was due, participants were provided details of the chief investigator for referral to a physiotherapist for assessment. Physiotherapists conducting injury assessments were not involved in baseline sprint testing, data processing, or statistical analysis.

### 2.3. HSI Reporting

Reporting HSI for cross-sectional analysis was based on self-report only. Although participants who reported a previous HSI largely had confirmation through medical professional diagnosis and/or imaging, this evidence was not individually sought. For cohort analysis, as mentioned above, a physiotherapist confirmed the HSI diagnosis. To be included in the study analysis, the HSI must have occurred while training or in competition during non-contact, high-speed running.

### 2.4. Data Processing

#### 2.4.1. IMU Data

IMU sprint trial data were exported into Matlab (The MathWorks Inc., Version R2020b, Natick, MA, USA) software for POC detection, which was performed using the vertical, earth-referenced accelerometer data (axial axis) from the shank IMU. The first negative minimum in the acceleration trace following maximal hip flexion was manually determined for each stride cycle. Anterior pelvic tilt and hip flexion angular data at the POC, as well as stride and step time, were subsequently exported into Excel (Microsoft Excel 2019, Microsoft Corporation, Washington, DC, USA). The shank sensor was only used for POC determination, as it was not accurate enough for use based on previous validation [33].

Six consecutive steps were selected to represent steady-state running for analysis of kinematic data in the latter part of the 40 m sprint. The root mean square error (RMSE) of the hip flexion data at the POC of every 6 consecutive steps was calculated, and the 6 steps with the lowest RMSE were selected (i.e., 3 steps on each side).

#### 2.4.2. FVP Data

Five-meter split times from the first 30 m of the sprint were calculated using the ‘MySprint’ iPhone application (Version 1.10 installed on iPhone XR running iOS 13). The start of the sprint was the first frame in which the participant’s thumb left the ground. Split times were determined by scrolling through the captured video using the application, aligning the participant’s hip with respective agility poles. Each split time was recorded in milliseconds.

Participant split times, height, and weight, as well as temperature and air pressure at the time of testing, were inputted into a preformatted, free-access Excel spreadsheet [40] (Microsoft Excel 2019, Microsoft Corporation, Washington, USA) using the ‘Solver’ add-in macro to implement validated FVP equations. The following FVP variables were automatically generated for each participant:

- 30 m time (s);
- Vmax: Maximum velocity (m/s);
- F0: Theoretical maximum horizontal force at null velocity (N);
- V0: Theoretical maximum velocity under zero load (m/s);
- Pmax: Maximal mechanical power output (W/kg);
- FV Slope: Linear F-V relationship slope, negative indicates more force-oriented, positive indicates more velocity-oriented (−1 ≥ 1);
- RFmax: Ratio of horizontal component of ground reaction force (RF) maximum value (%);
- Drf: Rate of decrease in RF (%);
- Vopt: Speed at maximal power output (m/s).

### 2.5. Statistical Analysis

#### 2.5.1. Cross-Sectional Analysis 

Cross-sectional data were analysed using SPSS Statistics (IBM SPSS Software, Version 15.0). Mixed Model Analysis was used to compare kinematic differences between groups in each of the IMU variables listed above. A covariance structure of autoregressive AR1 was used with the fixed factor of HSI either coded by side (i.e., coding each side as either with or without a history of HSI) or by participant (i.e., coding the participant as with or without a history of HSI), with the step number as repeated measures and participant ID as the subject identifier. Given the evidence of age and sex differences in sprinting performance [41], differences in FVP data between groups were analysed through Analysis of Covariance (ANCOVA), while controlling for age and sex.

#### 2.5.2. Cohort Analysis

The small sample size (as a result of restrictions imposed with COVID-19) precluded formal prospective statistical analysis. For those who sustained an HSI in the surveillance period, percentile evaluation was used to compare those who sustained an HSI in the surveillance period to controls for FVP variables and to compare injured sides to uninjured sides for IMU variables across the full cohort.

## 3. Results

### 3.1. Cross-Sectional Results

#### 3.1.1. IMU Data Findings

Five participants were removed because of unusable IMU data. Thus, 18 participants in total were included in the cross-sectional IMU data analysis. Eleven had a history of HSI (mean age 33.1 ± 11.8 years; height 1.73 ± 0.1 m; weight 70.2 ± 13.0 kg; eight females, three males); seven did not (mean age 19 ± 3.3 years; height 1.76 ± 0.1 cm; weight 72.8 ± 5.8 kg; two females, five males). Table 1 details the cross-sectional statistical analysis results for IMU data.

#### 3.1.2. FVP Data Findings

All participants (n = 23) were included in cross-sectional FVP data analysis. Thirteen had a history of HSI (mean age 31 ± 11.9 years; height 1.73 ± 0.1 m; weight 70 ± 12.7 kg; eight females, five males); ten did not (mean age 19 ± 2.7 years; height 1.78 ± 0.1 m; weight 74 ± 5.9 kg; two females, eight males). Baseline cross-sectional analysis revealed no significant differences in FVP between participants with and without a history of HSI. Age and sex covariates, however, influenced the FVP data as indicated in Table 2. Increasing age was associated with a slower 30 m time and reductions in Pmax and RFmax. For sex, males had faster 30 m times, as well as increased Vmax, F0, V0, Pmax, and RFmax.

### 3.2. Cohort Results

All participants completed the full surveillance period. Two male participants sustained an HSI of the high-speed running type during the one-year surveillance period (HSI#1, age: 24 years, height: 1.78 m, weight: 83 kg; HSI#2, age: 18 years, height: 1.75 m, weight: 77 kg). Both injuries were classified recurrent (not index), affecting the same side documented at baseline. No HSIs of the stretch type were sustained within the cohort. Percentile evaluation revealed the two participants who sustained an HSI were in the higher percentiles for maximum velocity (m/s) for both the entire cohort (HSI#1: 90th percentile, HSI#2: 85th percentile) and the group with a previous history of HSI (HSI#1: 91st percentile, HSI#2: 82nd percentile). There were IMU variables that seemed to be single outliers (e.g., reduced anterior pelvic tilt, step time, and stride time), but none that were consistent across both injured participants. All variable percentiles are detailed in Table 3.

## 4. Discussion

This study retrospectively and prospectively investigated the relationship between running biomechanics and HSI and reported preliminary findings that anterior pelvic tilt and FVP may be associated with HSI. Unfortunately, COVID-19 restrictions resulted in a small sample size, limiting the power and subsequent generalisability of the findings. Nevertheless, the protocol may be considered a feasibility trial of novel technology (IMUs and FVP using iPhone app), and the preliminary findings present a case for further investigation. In this section, we will discuss the main findings, as well as offer valuable lessons learnt to direct future observational research.

### 4.1. Anterior Pelvic Tilt and HSI

In our study, increased anterior pelvic tilt at the POC (on previously injured side) during sprinting was associated with HSI. This finding was at the cross-sectional level; thus, it cannot be determined whether this finding may be a cause or consequence of HSI. It is important to also acknowledge that for the cross-sectional arm of the study, HSI was based on retrospective self-report only. Our prospective cohort analysis of two participants who sustained an HSI did not support increased anterior tilt as a cause of HSI. Rather, one of these two participants actually scored in the lower percentiles for anterior pelvic tilt at the POC.

A compelling argument exists for how anterior pelvic tilt may play a role in HSI. The hamstring muscles (except the short head of the biceps femoris) originate from the ischial tuberosity within the pelvis; hence, theoretically, any level of pelvic tilt will affect hamstring length and subsequent tension loads [42]. Moreover, a time series analysis using video raster stereography demonstrated an inverse relationship between anterior pelvic tilt and hip and knee flexion in the late swing phase of high-speed running [43]. The authors proposed that increased anterior pelvic tilt would limit hip flexion, prompting the athlete to extend the knee further as a compensatory mechanism [43]. Although decreased hip flexion could be interpreted as protective [44,45], increased knee extension places a significant load on the hamstrings [46], as they must generate large eccentric forces to decelerate the knee joint and prepare the limb for contact [47]. Excessive muscle strain associated with this late-swing eccentric contraction is what is believed to be the primary mechanism behind HSIs of the ‘high-speed running’ type [6].

Our findings are comparable to other studies that have also investigated pelvic kinematics and HSI. On a retrospective level, Daly et al. (2016) found increased late-swing anterior pelvic tilt in participants with a history of HSI, when compared to controls [48]. Higashihara et al. (2019) reported decreased late stance anterior pelvic tilt on the injured side (compared to the uninjured side) [49], and Schuermans et al. (2017) found no difference in anterior pelvic tilt throughout the gait cycle between participants with and without a history of HSI [50]. Prospectively, Schuermans et al. (2017) reported increased anterior pelvic tilt in early swing in participants who sustained an HSI in their cohort study [50], while Kenneally-Dabrowski et al. (2019) found no difference in late-swing anterior pelvic tilt between cohort groups [51].

An important consideration when interpreting anterior pelvic tilt findings within and between studies is calibration protocol. Some studies (including the present study) calibrate in neutral standing posture, while others calibrate from true vertical. A recent study measured standing anterior pelvic tilt (relative to vertical) at baseline before a five-year HSI surveillance period in football players. They reported increased neutral standing anterior pelvic tilt in those who sustained an HSI during the surveillance period [52]. The current study calibrated zero in neutral standing posture, so running measurements reflected only the angular deviation from standing.

Owing to variations in running protocols and discrepancies in the running phases analysed and calibration protocol, the ability to draw definitive conclusions across these studies is limited. Nevertheless, there is a case for further investigation into the association between anterior pelvic tilt and HSI. Future studies should consider reporting on anterior pelvic tilt in neutral standing posture and then documenting the relative change in running in order to direct the development of relevant preventative strategies, e.g., target static lumbopelvic control and/or dynamic lumbopelvic control.

### 4.2. FVP and HSI

Another preliminary finding from our study, warranting further investigation, is the difference in FVP between participants who prospectively sustained an HSI and the controls. While our retrospective analysis revealed no difference in FVP between participants with and without a history of HSI, prospective analysis of two participants revealed both participants scored in the higher percentiles for variables associated with velocity (Vmax, V0, and Vopt). One of these participants also rated in high percentiles for horizontal force propulsion (F0: 85%), power output (Pmax: 100%), and mechanical effectiveness measured by the rate of decline in reaction force with increasing speed (Drf: 100%). Our findings bear similarities to a cross-sectional study that also utilised the ‘MySprint’ iPhone application, reporting higher V0 and Drf in sub-elite male football players who had a history of HSI, when compared to controls [53].

Other studies found no significant differences in velocity-related variables but reported an association between low F0 and HSI. A large prospective cohort study used radar or laser technology for sprint velocity data to compute FVP over two 30 m sprints in university and professional football players at different time points during the season. They reported that lower F0 values were significantly associated with HSI risk in the weeks following the sprint measurement [12]. A case–control study utilising radar technology for FVP also found reduced F0 (as well as Pmax) in semi-professional football players compared to controls. Testing was undertaken immediately after clearance of return to play following HSI, and nil difference between groups was reported upon testing 2 months after [36]. The same research group later published a paper of two HSI case reports: one soccer player with FVP data immediately before and during the injury, and one rugby player with FVP data seven days before HSI and after return to sport. They found that the horizontal force was reduced before and after the respective HSIs [11]. Finally, a prospective cohort study recorded only F0 using radar technology for FVP over two 30 m sprints. They reported an association between lower F0 and HSI occurring between pre- and mid-season in premier division male Finnish football players [14]. The absence of velocity-related data limits the comparability of findings. Nevertheless, the findings of our study and those aforementioned above present emerging evidence for a relationship between sprint mechanical output and HSI. Ultimately, more research is needed to better understand normative data and clarify which FVP variables are of relevance to HSI for varying sports (or field positions), demographics, and stages of injury (e.g., for primary, secondary, or tertiary prevention).

For better research translation, future studies may consider adopting the FVP classification method presented by Hicks and colleagues [54]. Their classification system explains the interaction between velocity and force-orientated profiles by categorising FVP into four quadrants: 1: high F0 and V0, 2: high F0 and low V0, 3: low relative F0 and V0, 4: low F0 and high V0. Thresholds should be determined relative to the specific cohort. For each quadrant, they propose the effect on sprint performance (initial steps versus upright maximal velocity), including technical characteristics (e.g., level of forward lean) and suggested training recommendations to enhance performance [54]. If future epidemiological studies determine these FVP quadrants are relevant in the context of HSI, Hicks et al.’s training recommendations may be a good starting point for interventional studies [54].

### 4.3. Direction for Future Studies

#### 4.3.1. Use of IMUs

Several lessons learnt from this study regarding the use of IMUs can be shared. Although our IMU data were validated [33], and in agreement with other studies involving overground sprinting [55,56,57,58,59], there were data gaps in five participants resulting in exclusion from analysis. Noraxon’s newer version ‘Ultium’ provides in-sensor data storage which may mitigate this issue by filling gaps where live transmission is interrupted [60]. Nonetheless, several factors may have contributed to the incompleteness of these data sets, particularly those related to field-based usage.

The IMU system was validated in a laboratory environment against a gold standard motion analysis system [33]. In this setting, a variety of common IMU issues can be controlled. Using IMUs in the field presents various concerns, including power supply problems [29], sensor noise [61], synchronisation issues [62], magnetic disturbances [63], and environmental interferences [19], all of which may contribute to the intermittent cessation of recording leading to missing data. Furthermore, the validation was conducted on a motorised treadmill with a controlled speed, slope, and surface, all of which are variables that cannot be controlled in field-based, overground conditions.

For future studies using IMUs for running, we recommend several mitigation strategies to address these issues. Firstly, data should be validated in environments that closely replicate real-world conditions, e.g., in overground conditions or outside the laboratory (where feasible). Guidelines and a standardised measurement process for IMUs are needed to enhance the accuracy of running measurements and improve comparability across studies [21]. Although high sampling rates are advised for a greater accuracy of high-speed speeding analysis [64], they can result in data overload. Thus, it is advisable to minimise the number of sensors used [65]. On testing day, check for local magnetic disturbance and delay/move tests accordingly [55], or consider developing a magnetometer-free method [20]. As for calibration, the procedure for overground running should incorporate assumptions to simplify the global reference frame of the IMU to minimise drift errors [65]. Finally, a full battery charge should always be ensured.

#### 4.3.2. Protocol Considerations

Upon completion and reflection of this study, key takeaways can be offered in terms of study design and methodology to enhance data reliability and generalisability. On top of having a prospective cohort design and large sample size, future studies should test over several time periods, quantify exposure, and extend running data capture distance.

In real-life conditions, the assessment of running biomechanics may be considered as part of musculoskeletal screening for both injury risk reduction and performance [66]. Time and costing constraints can limit musculoskeletal screening to only one occasion at baseline, but in reality, screening should be conducted regularly as results can fluctuate throughout the season [67]. Variables such as isometric hamstring strength have been used as an in-season monitoring secondary prevention tool for HSI, with testing after every match informing training volume for the subsequent week [68]. Indeed, this level of testing frequency is currently not feasible for running biomechanical analysis due to longer setup and processing time. However, future technological advances may permit simultaneous running analysis within competition and training, e.g., through validation of vision machine learning methods [17,69]. In the meantime, it is recommended to test throughout the surveillance period as often as practicable.

The collection of exposure data may also help us understand potential fluctuations in results throughout the season and other confounding factors for injury. In sports performance, the term “exposure” commonly refers to the intensity and duration of physical activity, which together contribute to an athlete’s overall workload. It encompasses the time spent in training or competition and can include a detailed breakdown of specific tasks performed [70]. Sudden increases in high-speed running can increase HSI risk [3], and equally, not enough high-speed conditioning can increase HSI risk [4]. An explanation for this disparity in findings is likely related to fatigue, and that a certain level of high-speed running load may be protective in nature [71,72].

Finally, it may be worth extending the running data capture distance. Our testing distance was 30 m for FVP, with an additional 10 m for kinematic data capture (40 m in total). This may be sufficient for many multi-directional team sports, where the average sprint effort is less than 30 m [73], but may be insufficient for track sprinters who compete over distances of 100 m or greater. Furthermore, as a result of IMU validation constraints, we only captured sagittal plane pelvis and hip kinematic data at the point of contact in upright running. Future studies may look to investigate whether there is a link between kinematics in the early phases of acceleration [74], force–velocity profile, and HSI.

## 5. Conclusions

This combined cross-sectional and prospective cohort study demonstrated preliminary findings that present a case for further research investigating running biomechanics and HSI. Cross-sectional analysis revealed participants with a history of HSI exhibited increased anterior pelvic tilt at the point of contact (on the injured side) during upright sprinting when compared to controls. Prospectively, participants who sustained an HSI scored in the higher percentiles for variables associated with a velocity-oriented profile (Vmax, V0, and Vopt). The sample size was limited; thus, the findings should be interpreted for feasibility purposes only. Therefore, several practical insights were suggested for the use of novel technology (IMU and FVP) to quantify running biomechanics for future observational research, which ultimately aims to inform HSI prevention strategies.

## Figures and Tables

**Table 1:** Cross-sectional analysis of IMU data by HSI side and by subject, using Mixed Model Analysis (Type III Fixed Effects).

IMU Variable	HSI History by Side μ (95% CI)	Control by Side μ (95% CI)	Sig. by Side (*p* Value)	HSI History by Participant μ (95% CI)	Control by Participant μ (95% CI)	Sig. by Participant (*p* Value)
Anterior Pelvic Tilt at POC (^ο^)	3.1 (−0.2, 6.4)	1.7 (−1.6, 5.0)	<0.001 *	2.3 (−1.4, 7.1)	1.0 (−4.3, 6.3)	0.573
Hip Flexion at POC (^ο^)	56.7 (52.4, 61.1)	55.2 (51.0, 59.3)	0.121	53.8 (48.6, 59.0)	58.7 (52.2, 65.2)	0.231
Step time (s)	0.238 (0.231, 0.244)	0.237 (0.232, 0.242)	0.796	0.24 (0.235, 0.246)	0.232 (0.225, 0.239)	0.053
Stride time (s)	0.473 (0.461, 0.485)	0.474 (0.462, 0.486)	0.478	0.48 (0.464, 0.495)	0.465 (0.445, 0.484)	0.216

IMU: inertial measurement unit, HSI: hamstring strain injury, μ: mean, CI: confidence interval, POC: point of contact, * significant difference between groups.

**Table 2:** Cross-sectional analysis of FVP data by subject, using Analysis of Covariance, controlling for age and sex.

FVP Variable	HSI History by Side μ (SD)	Control by Side μ (SD)	*p* Value
30 m Time (s)	4.87 (0.36)	4.60 (0.24)	0.463 *^
Vmax (m/s)	7.48 (0.81)	8.01 (0.59)	0.660 ^
F0 (N/kg)	8.98 (1.28)	8.90 (0.48)	0.493 ^
V0 (m/s)	7.77 (0.93)	8.45 (0.70)	0.663 ^
Pmax (W/kg)	17.39 (2.97)	18.80 (1.63)	0.312 *^
FV Slope	−1.75 (0.24)	−1.06 (0.12)	0.753
RFmax (%)	44.55 (2.98)	46.56 (1.75)	0.582 *^
Drf (%)	−10.8 (0.9)	−10.5 (0.6)	0.782
Vopt (m/s)	4.01 (0.10)	3.92 (0.14)	0.664

FVP: force–velocity profile, HSI: hamstring strain injury, μ: mean, SD: standard deviation, Vmax: maximum velocity, F0: theoretical maximum horizontal force at null velocity, V0: theoretical maximum velocity under zero load, Pmax: maximal mechanical power output, FV Slope: linear F-V relationship slope (negative indicates more force-oriented, positive indicates more velocity-oriented), RFmax: ratio of horizontal component of ground reaction force (RF) maximum value, Drf: rate of decrease in RF, Vopt: speed at maximal power output, * significant difference in age covariant between groups, ^ significant difference in sex covariant between groups.

**Table 3:** Cohort baseline variable percentiles for the two participants who sustained an injury during the one-year surveillance period.

	Variable	HSI Cohort Participant #1 Percentile of Cohort	HSI Cohort Participant #2 Percentile of Cohort
IMU Data	Mean Anterior Pelvic Tilt at POC—Injured Side	14.9%	17.4%
	Mean Anterior Pelvic Tilt at POC—Uninjured Side	0.9%	32.9%
	Mean Hip Flexion at POC —Injured Side	81.3%	14.3%
	Mean Hip Flexion at POC —Uninjured Side	67.9%	61.6%
	Step Time—Injured Side	0.0%	19.7%
	Step Time—Uninjured Side	77.5%	4.2%
	Stride time—Injured Side	16.2%	2.1%
	Stride time—Uninjured Side	22.5%	2.8%
FVP Data	30 m Time	20.0%	5.0%
	Vmax	90.0%	85.0%
	F0	25.0%	85.0%
	V0	90.0%	85.0%
	Pmax	55.0%	100.0%
	FV Slope	90.0%	50.0%
	RFmax	65.0%	100.0%
	Drf	90.0%	55.0%
	Vopt	90.0%	85.0%

IMU: inertial measurement unit, FVP: force–velocity profile, HSI: hamstring strain injury, μ: mean, SD: standard deviation, Vmax: maximum velocity, F0: theoretical maximum horizontal force at null velocity, V0: theoretical maximum velocity under zero load, Pmax: maximal mechanical power output, FV Slope: linear F-V relationship slope (negative indicates more force-oriented, positive indicates more velocity-oriented), RFmax: ratio of horizontal component of ground reaction force (RF) maximum value, Drf: rate of decrease in RF, Vopt: speed at maximal power output.

## Data Availability

Data are available from the authors upon reasonable request.
